# Physiological and Transcriptomic Analysis Reveals That Melatonin Alleviates Aluminum Toxicity in Alfalfa (*Medicago sativa* L.)

**DOI:** 10.3390/ijms242417221

**Published:** 2023-12-07

**Authors:** Congge Liu, Haijing Cheng, Shuwei Wang, Dashi Yu, Yunmin Wei

**Affiliations:** College of Life Sciences and Oceanography, Shenzhen University, Shenzhen 518060, China; conggeliu93@163.com (C.L.); 13644654490@163.com (H.C.); shuweiwang_21@163.com (S.W.)

**Keywords:** Al toxicity, melatonin, redox homeostasis, RNA sequencing, cell wall, carbon metabolism

## Abstract

Aluminum (Al) toxicity is the most common factor limiting the growth of alfalfa in acidic soil conditions. Melatonin (MT), a significant pleiotropic molecule present in both plants and animals, has shown promise in mitigating Al toxicity in various plant species. This study aims to elucidate the underlying mechanism by which melatonin alleviates Al toxicity in alfalfa through a combined physiological and transcriptomic analysis. The results reveal that the addition of 5 μM melatonin significantly increased alfalfa root length by 48% and fresh weight by 45.4% compared to aluminum treatment alone. Moreover, the 5 μM melatonin application partially restored the enlarged and irregular cell shape induced by aluminum treatment, resulting in a relatively compact arrangement of alfalfa root cells. Moreover, MT application reduces Al accumulation in alfalfa roots and shoots by 28.6% and 27.6%, respectively. Additionally, MT plays a crucial role in scavenging Al-induced excess H_2_O_2_ by enhancing the activities of superoxide dismutase (SOD), peroxidase (POD), and catalase (CAT), consequently reducing malondialdehyde (MDA) levels. More interestingly, the RNA-seq results reveal that MT application significantly upregulates the expression of *xyloglucan endotransglucosylase/hydrolase* (*XTH*) and carbon metabolism-related genes, including those involved in the glycolysis process, as well as sucrose and starch metabolism, suggesting that MT application may mitigate Al toxicity by facilitating the binding of Al to the cell walls, thereby reducing intracellular Al accumulation, and improving respiration and the content of sucrose and trehalose. Taken together, our study demonstrates that MT alleviates Al toxicity in alfalfa by reducing Al accumulation and restoring redox homeostasis. These RNA-seq results suggest that the alleviation of Al toxicity by MT may occur through its influence on cell wall composition and carbon metabolism. This research advances our understanding of the mechanisms underlying MT’s effectiveness in mitigating Al toxicity, providing a clear direction for our future investigations into the underlying mechanisms by which MT alleviates Al toxicity in alfalfa.

## 1. Introduction

It is estimated that approximately 40–50% of the world’s arable lands had become acidic by the year 2015, with nearly 60% of these acidic lands concentrated in tropical and subtropical regions, which are the primary grain-producing areas [1]. Recent societal developments have exacerbated soil acidification due to anthropogenic factors such as the indiscriminate application of synthetic fertilizers, the accumulation of organic matter, and disruptions in the natural nutrient cycling within the soil [2]. Aluminum (Al), the third most abundant element in the Earth’s crust, assumes a paramount role in this effect as it represents the most crucial and widespread factor limiting crop yields in acidic soils. In such acidic environments, aluminum dissolves into a toxic ionic form, notably Al^3+^, originating from less harmful aluminosilicates or aluminum oxides [3].

Numerous studies have indicated that the primary symptoms of Al toxicity in plants include inhibited root growth and biomass, resulting in altered root morphology [4,5]. The root apical transition zone (TZ), located between the meristem and elongation zone, is particularly sensitive to Al exposure [5,6,7,8,9]. Another significant consequence of Al toxicity in plants is the rapid production and accumulation of reactive oxygen species (ROS), such as hydrogen peroxide (H_2_O_2_), superoxide (O_2_^·−^), and hydroxyl (OH^·−^) radicals. This ROS buildup leads to membrane lipid peroxidation, intensifying damage to the membrane system, protein degradation, and ultimately programmed cell death [5,10,11,12]. To counteract the excessive ROS accumulation induced by Al, plants activate an enzyme-mediated antioxidant defense system, which includes superoxide dismutase (SOD), ascorbate peroxidase (APX), catalase (CAT), and peroxidases (POD) [13,14]. SOD plays a pivotal role in the oxidative stress response by catalyzing the dismutation of O2^·−^ into oxygen and H_2_O_2_, marking the initial step in this process, after which H_2_O_2_ is further converted into water through the catalytic actions of APX, CAT, and POD [15]. Furthermore, non-enzymatic antioxidants, including proline, ascorbate, and glutathione, have been documented to effectively detoxify ROS and enhance Al tolerance [16,17], whose production and activation are integral components of the internal tolerance mechanism that plants employ to combat Al toxicity. Additionally, plants combat Al toxicity through intracellular Al chelation and the compartmentalization of Al-organic acid complexes within vacuoles [5,18,19]. Beyond the internal mechanisms, Al exclusion mechanisms have received substantial attention in research. These mechanisms involve the secretion of organic acids (OAs) as a well-characterized and extensively studied response to Al stress, including citrate, malate, and oxalic acids, which serve to chelate Al and form non-toxic complexes [1,5]. Citrate and malate are the main types of OA secreted in many plant species. For example, crops such as rice [20], maize [21], and pea [22] have been shown to release citrate in response to Al stress. Malate secretion has been identified in plants like rape [23] and sorghum [24]. Additionally, some species, including *Arabidopsis* [25], soybean [26], and barley [27], can secrete both citrate and malate when confronted with Al stress. In contrast, oxalic acid secretion has been detected in buckwheat and taro, with no known transporters identified for its secretion in response to Al toxicity. Numerous experiments have pinpointed the involvement of families such as multidrug and toxic compound extrusion (MATE), responsible for encoding citrate transporters on the plasma membrane, and Al-activated malate transporter (ALMT), an anion channel activated by Al^3+^ on the plasma membrane, in facilitating the release of the respective OAs from plant root cells into the rhizosphere under Al stress [20,21,22,23,24,25,26,27]. However, transporters for oxalic acid secretion in response to Al toxicity remain unidentified. In certain Al-tolerant species like *Melastoma malabathricum* and *Melaleuca cajuputi*, in addition to exuding OAs for Al chelation, they also produce phenolic compounds in their roots that can chelate Al [17]. Hence, the extent of Al tolerance varies among different plant species. Alfalfa (*Medicago sativa* L.), a widely cultivated forage crop renowned for its nutritional value and high yield capacity, is highly sensitive to Al stress and has been associated with severely curtailing alfalfa productivity in acidic soils [28]. Therefore, it is important to investigate alfalfa’s response to Al stress and adopt appropriate strategies to enhance alfalfa yield.

Melatonin (N-acetyl-5-methoxytryptamine) is an indole derivative that was initially discovered in vascular plants in 1995 [29]. It has been identified as a key regulator in various physiological processes, including the regulation of circadian rhythms [30], the promotion of seed germination, root development, enhancements in photosynthesis [31], and the postponement of leaf and flower senescence [32]. One of the most extensively characterized and studied roles of melatonin is its ability to enhance plant tolerance to various abiotic stresses. For instance, exogenous melatonin has been shown to improve salt tolerance in sunflower [33] and rice [34], it plays a crucial role in mitigating the toxicity of heavy metals, and it alleviates cadmium toxicity in Chinese cabbage seedlings [35], chromium toxicity in maize [36], and copper toxicity in cucumber [37]. Researchers have also explored the potential alleviating effects of melatonin on Al toxicity. Its application has been found to enhance resistance to Al toxicity by increasing the antioxidant defense system and promoting the exudation of Al-induced malate and citrate in soybean [38] and wheat [39]. Ren et al. [40] demonstrated that melatonin mitigates Al-induced growth inhibition in maize [41] by modulating carbon and nitrogen metabolism and regulating redox homeostasis. However, it remains poorly understood whether melatonin can alleviate the inhibitory effects of Al treatment on alfalfa, and the molecular mechanisms underlying how melatonin mitigates Al-induced growth inhibition in alfalfa have not been adequately elucidated.

In recent years, RNA sequencing (RNA-seq) has emerged as an indispensable tool for transcriptome analysis because it can generate a vast number of reads, providing a comprehensive overview of the transcriptomic landscape. Consequently, RNA sequencing has been applied to investigate Al response mechanisms in various plant species, including *Pinus massoniana* [42], sugarcane [43], aspen [44], peanut [45], and alfalfa [46], uncovering numerous physiological and metabolic processes involved in Al stress such as oxidative stress, organic acid exudation, defense against cell wall toxicity, and hormone signal transduction. Despite extensive information on Al stress responses, the specific mechanisms via which melatonin regulates Al toxicity in alfalfa remain unexplored. In our study, we integrate physiological and transcriptomic analyses to investigate the regulatory mechanisms through which melatonin might mitigate Al toxicity in alfalfa and provide valuable insights into a theoretical foundation for enhancing alfalfa yield in acidic soil conditions.

## 2. Results

### 2.1. Melatonin Applications Significantly Alleviate Al-Induced Growth Inhibition in Alfalfa

**Figure 1:**
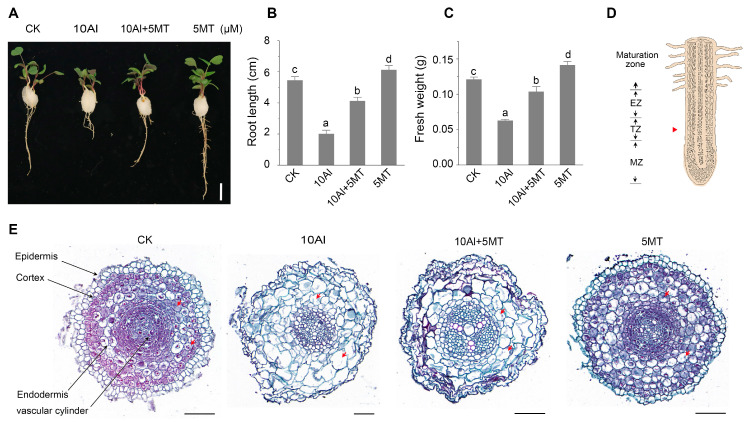
Melatonin alleviates aluminum-induced phenotypic changes in alfalfa. (**A**) Phenotypic comparison after 3 days of AlCl_3_ and melatonin treatment. Scale bar, 1 cm. (**B**) Alfalfa root length; data are shown as mean ± SE (n = 16). (**C**) Fresh weight of four alfalfa seedlings on a cotton ball; data are shown as mean ± SE (n = 4). Tukey’s test was used to determine the statistical significance, marked with lowercase letters at the level of *p* ≤ 0.05. (**D**) The cross-sectional site (red arrow) of the root tip, meristematic zone (MZ), transition zone (TZ), and elongation zone (EZ). (**E**) Cross-sectional view of alfalfa root tips cut in the middle of the transition zone shown in (**D**), using safranin and fast green dyes for staining. The cells indicated by the red arrows represent significant changes in the size and shape of cortical cells after different treatments. Scale bar, 20 μm.

Previous research has demonstrated that the optimal concentration of exogenous melatonin required to mitigate Al toxicity varies among different plant species. For instance, 1 μM exogenous melatonin is most effective for soybeans [38], while 50 μM exogenous melatonin is optimal for maize [41]. To determine the optimal concentration of exogenous melatonin for alleviating Al toxicity in alfalfa, we conducted an experiment in which we observed the growth of alfalfa under different melatonin concentrations (1 μM, 5 μM, 10 μM, and 50 μM) while exposed to 10 μM AlCl_3_. We assessed root length and fresh weight as indicators of plant growth and found that melatonin significantly mitigates Al toxicity, which was most pronounced at lower melatonin concentrations (1 μM and 5 μM) but diminished at higher concentrations (10 μM and 50 μM) (Supplementary Figure S1). Thus, 5 μM melatonin was selected for further investigation as it exhibited the most effective alleviation of Al toxicity (Figure 1A).

In the absence of melatonin, exposure to 10 μM AlCl_3_ resulted in a substantial reduction of 41.2% in alfalfa root length and a 33.3% decrease in fresh weight. However, 5 μM melatonin showed a remarkable improvement, with alfalfa exhibiting a 48% increase in root length and a 45.4% increase in fresh weight (Figure 1B,C). Importantly, the primary symptoms of Al toxicity extend beyond the inhibition of root growth and encompass disruptions in root morphology [4,5]. To assess cell morphology, transverse sections of the root tip cells were examined. The root apical cells in the transition zone exhibited a normal rectangular shape with a tight arrangement without Al treatment. However, the cells became enlarged and assumed irregular shapes with a loose arrangement under Al stress. Excitingly, the addition of melatonin during Al treatment effectively maintained the root’s cellular morphology (Figure 1E and Figure S3). These results collectively demonstrate that the application of exogenous melatonin effectively alleviates Al-induced growth inhibition and preserves the morphology of root tip cells in alfalfa.

### 2.2. Melatonin Could Reduce the Accumulation of Al under Al Stress in Alfalfa

**Figure 2:**
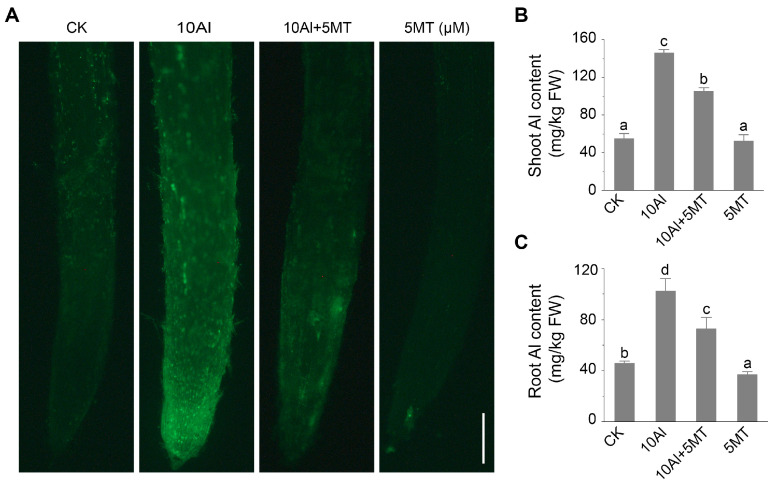
Effects of melatonin on Al accumulation in alfalfa roots. (**A**) Morin staining after 3 days of AlCl_3_ and melatonin treatment. Scale bar, 100 μm. The Al content in alfalfa shoot (**B**) and root (**C**) after 3 days of AlCl_3_ and melatonin treatment. Data are shown as mean ± SD (n = 3), different letters are significantly different (*p* ≤ 0.05) according to Tukey’s test.

To investigate the mechanism by which melatonin alleviated Al toxicity in alfalfa, the Al content of alfalfa seedings treated with and without melatonin for 3 days was qualitatively and quantitatively analyzed. Morin staining, a sensitive fluorescent dye for Al, was used to visualize Al presence in the roots. The green fluorescence of morin was only faintly detected without Al treatment, whereas after exposure, Al-treated alfalfa tips displayed stronger Al-dependent green fluorescence than those treated with Al combined with melatonin. (Figure 2A). These findings were further supported by a quantitative analysis of Al content. Exogenous melatonin treatment reduced Al accumulation in alfalfa roots and shoots by 28.6% and 27.6%, respectively, compared to Al treatment alone (Figure 2B,C). These results confirmed that melatonin mitigates Al toxicity in alfalfa by reducing Al content.

### 2.3. Melatonin Could Alleviate Al-Induced Oxidative Damage in Root Cells

**Figure 3:**
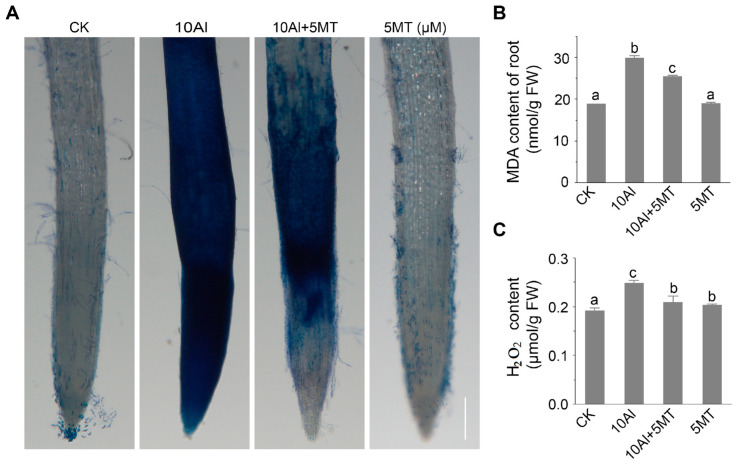
Effects of melatonin on Al-induced oxidative damage in alfalfa roots. (**A**) Evans blue staining after 3 days of AlCl_3_ and melatonin treatment. Scale bar, 100 μm. The content of MDA (**B**) and H_2_O_2_ (**C**) in alfalfa root after 3 days of treatment. Data are shown as mean ± SD (n = 3), different letters are significantly different (*p* ≤ 0.05) according to Tukey’s test.

Melatonin is well-known for its ability to enhance plant resistance to various abiotic stresses, including heavy metal stress [35,36,37], drought [41], and salt stress [34], primarily due to its potent free radical scavenging capabilities. Therefore, we sought to validate the mitigating effects of melatonin on cellular oxidative damage by assessing H_2_O_2_ levels, malondialdehyde (MDA) content, and root cell vitality, which are indicative of Al-induced oxidative damage. The application of melatonin significantly reduced Al-induced H_2_O_2_ levels by 15.7%, although they did not return to control levels, compared to the H_2_O_2_ content treated only with Al (Figure 3B). Furthermore, it is known that the accumulation of free radicals in plants can result in membrane lipid peroxidation. MDA, a final product of lipid peroxidation, is positively correlated with the extent of this peroxidation. The result showed that the addition of melatonin reduced the significant accumulation of MDA induced by Al by 16.8%, indicating that melatonin applications could mitigate Al-induced lipid peroxidation in alfalfa roots (Figure 3C). Additionally, we evaluated root cell vitality using Evans blue staining, which accumulates in dying and dead cells. In alfalfa treated with Al alone, root cells displayed extensive blue patches, indicative of a large number of dying or dead cells. However, simultaneous treatment with melatonin and Al significantly reduced the number of dead cells, resulting in reduced blue staining (Figure 3A). These findings collectively suggest that melatonin can partially alleviate Al-induced oxidative damage in alfalfa root cells by reducing H_2_O_2_ levels, mitigating lipid peroxidation, and preserving membrane integrity.

### 2.4. Melatonin Could Enhance the Activity of Antioxidant Enzymes

**Figure 4:**
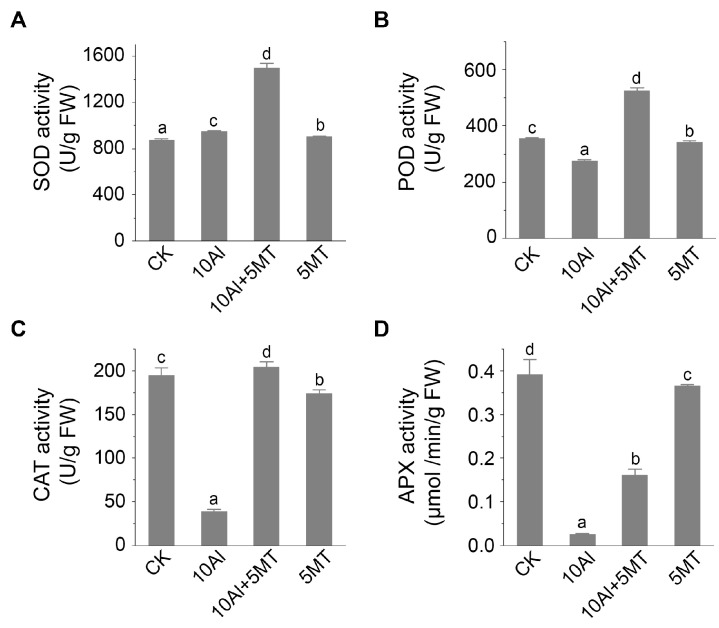
Effects of Al and melatonin on the activity of antioxidant enzymes in alfalfa roots. (**A**) Superoxide dismutase (SOD) activity, (**B**) peroxidase (POD) activity, (**C**) catalase (CAT) activity, and (**D**) ascorbate peroxidase (APX) activity in alfalfa roots after 3 days of treatment. Data are shown as mean ± SD (n = 3), different letters represent significant difference (*p* ≤ 0.05) according to Tukey’s test.

The primary strategy for mitigating oxidative damage involves the elimination of ROS through the action of antioxidant enzymes. We hypothesized that melatonin could enhance the activity of these antioxidant enzymes, thus maintaining ROS homeostasis. Thus, we assessed the activities of SOD, POD, CAT, and APX. The results showed that, compared to the control, Al treatment alone had minimal impact on SOD activity but reduced POD activity by 22.8%. Furthermore, it significantly inhibited the activities of CAT and APX in the roots. This inhibition of POD, CAT, and APX activities led to the accumulation of H_2_O_2_, which could not be effectively catalyzed into H_2_O and O_2_, consistent with our previous findings (Figure 3). Conversely, melatonin application, when compared to Al treatment alone, resulted in a substantial increase in the activities of SOD (approximately 1.58-fold), POD (approximately 1.91-fold), CAT (approximately 5.27-fold), and APX (approximately 5.27-fold), indicating that melatonin application could significantly enhance the ROS clearance capacity of alfalfa. Moreover, there was no significant difference in the activity of these enzymes between the control and melatonin-treated plants in the roots (Figure 4). Overall, these data suggest that melatonin application could effectively maintain ROS homeostasis by increasing the activity of antioxidant enzymes in alfalfa.

### 2.5. Treatments with Al and Melatonin Trigger Transcriptome Reprogramming in Alfalfa Roots

**Figure 5:**
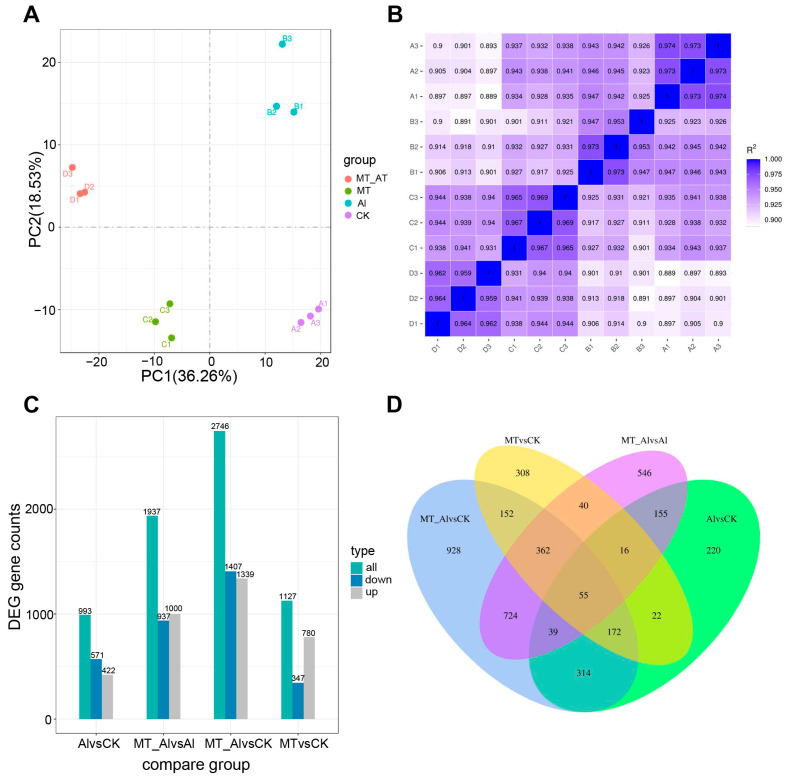
Global analysis of the transcriptome data of alfalfa roots. (**A**) Principal component analysis (PCA) of the RNA-seq data. (**B**) Correlation heatmap among the samples, with the horizontal and vertical coordinates representing the square of the correlation coefficients for each sample. A square of 1 for the correlation coefficient in the figure (as indicated by the dark blue color) represents the comparison of a sample with itself. The closer the square of the correlation coefficient is to 1, the higher the similarity between the patterns of expression among replicate samples. (**C**) Number of differentially expressed genes (DEGs) for each comparison. (**D**) Venn diagram illustrating DEGs in different comparisons. MT and Al represent the treatment of Al and melatonin (MT).

To explore the mechanisms underlying melatonin’s alleviation of Al toxicity, we conducted transcriptome analysis on alfalfa roots exposed to Al and Al + MT (melatonin) treatments, each with three biological replicates. Control (CK) and MT treatments were performed as experimental controls. Principal component analysis (PCA) and a heatmap of sample correlations (Figure 5A,B) indicated strong correlations among the biological replicates, affirming data accuracy and reliability. Then, we applied statistical analysis with criteria of |log2fold change| ≥ 1 and padj (Benjamini and Hochberg-adjusted *p*-values) ≤ 0.05 to identify DEGs in each comparison, which showed that, when comparing Al vs. CK, 993 DEGs were identified, comprising 422 upregulated and 571 downregulated genes. In the Al + MT vs. Al comparison, 1937 DEGs were detected, with 1000 upregulated and 937 downregulated genes (Figure 5C). The Venn diagram shows the number of DEGs among different treatment comparisons and highlights common and unique DEGs in each comparison. The results indicate that, upon comparing Al + MT vs. Al and Al vs. CK, 265 DEGs were found to be shared between them. Furthermore, the Al + MT vs. Al comparison revealed 1672 DEGs unique to it, while the Al vs. CK comparison had 728 exclusive DEGs (Figure 5D). These findings suggest that the addition of melatonin elicited the differential expression of a broader set of genes in response to Al stress.

### 2.6. Gene Ontology (GO) Classification and KEGG Pathway Analysis

**Figure 6:**
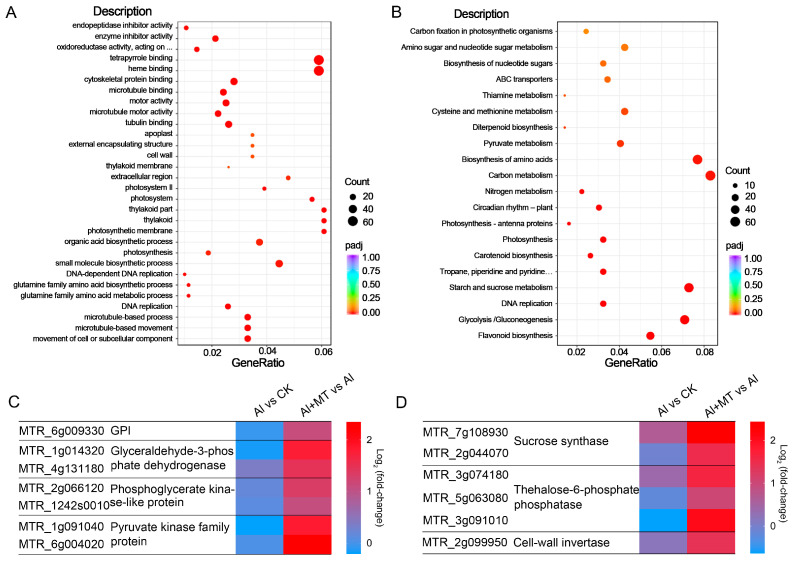
Enrichment analysis of differentially expressed genes (DEGs) on GO terms and KEGG pathways. (**A**) GO term enrichment analysis of the Al + MT vs. Al pair, showing the top 30 terms. (**B**) KEGG pathway enrichment analysis of the Al + MT vs. Al pair, showing the top 20 pathways. Count represents the number of genes associated with the specific function or pathway. The larger the red circle, the greater the number; conversely, the smaller the circle, the fewer the genes. (**C**) Transcript changes in glycolysis-related genes. (**D**) Transcript changes in starch and sucrose metabolism-related genes.

To determine the functions of these DEGs, we conducted gene ontology (GO) enrichment analysis with a threshold of padj (Benjamini and Hochberg-adjusted *p*-values) < 0.05. In the Al + MT vs. Al comparisons, the GO analysis categorized the DEGs into three domains: 380 biological process (BP) categories, 82 cellular component (CC) categories, and 261 molecular function (MF) categories. In the BP category, the DEGs were primarily associated with processes such as organic acid biosynthetic processes, cell wall organization or biogenesis, and the movement of cells or subcellular components. Concerning CC, a substantial proportion of DEGs were linked to the extracellular region and cell walls. In the MF category, the DEGs were predominantly related to activities involving oxidoreductase and microtubule motor functions (Figure 6A). These results suggest that the supplementation of exogenous melatonin might alleviate Al toxicity by not only mitigating Al-induced oxidative damage but also potentially regulating processes such as organic acid synthesis, cell wall organization, and cellular movement and remodeling as mechanisms for mitigating Al toxicity. Additionally, we conducted a Kyoto Encyclopedia of Genes and Genomes (KEGG) enrichment analysis to identify metabolic pathways associated with the effects of melatonin on Al stress in alfalfa. In the Al + MT vs. Al comparisons, the KEGG enrichment analysis revealed that these DEGs were predominantly enriched in metabolic pathways such as carbon metabolism, glycolysis/gluconeogenesis, and starch and sucrose metabolism (Figure 6B).

In the carbon metabolism and glycolysis/gluconeogenesis, most DEGs that were associated with glycolysis exhibited notable upregulation in Al + MT-treated plants (Figure 6C). These upregulated genes included those encoding pyruvate kinase, glyceraldehyde-3-phosphate dehydrogenase, and phosphoglycerate kinase-like protein, confirming that melatonin supplementation might enhance carbon metabolism in alfalfa roots under Al stress. In regard to pathways related to starch and sucrose metabolism, numerous DEGs annotated as encoding sucrose synthase, trehalose-6-phosphate phosphatase, and cell-wall invertase showed significant upregulation in the Al + MT vs. Al comparison (Figure 6D). These results suggest that the application of exogenous melatonin may boost the synthesis capacity of sucrose and trehalose in alfalfa, thereby enhancing their stress resistance under Al stress.

## 3. Discussion

In acidic soils, Al stress can significantly hinder plant growth, resulting in reduced crop yields, particularly in Al-sensitive crops such as alfalfa and soybean. Melatonin, an endogenous molecule with high conservation across eukaryotic evolution, plays a pivotal role as a signaling molecule in various biological processes and responses to abiotic stress in plants. These stresses include salt stress [33,34], drought stress [47], and heavy metal stress in certain species [35,36,37]. Recent studies have also revealed that melatonin mitigates Al toxicity in soybean [38], wheat [39], and maize [40,41] through diverse mechanisms. Our present study mainly investigated the mechanisms underlying the application of exogenous melatonin in alleviating Al stress in alfalfa.

### 3.1. Exogenous Melatonin Reduced Al Concentration in Both Alfalfa Roots and Shoots

It was reported that melatonin modified the polysaccharide content of cell walls, thereby reducing Al accumulation in the roots in wheat [39]. In line with these findings, our study showed that the application of exogenous melatonin significantly reduced Al content in both alfalfa roots and shoots under aluminum stress (Figure 2). Additionally, melatonin mitigated the growth inhibition of alfalfa induced by aluminum treatment (Figure 1). Next, we investigated whether melatonin reduced aluminum accumulation through modifying the polysaccharide content of cell walls. 

The cell wall, a dynamic architectural structure primarily comprising cellulose, hemicellulose, and pectin, plays a crucial role in sensing and responding to Al toxicity [1]. When plant roots are exposed to Al, the cell wall, serving as the initial point of contact, acts as a significant reservoir for Al. For instance, nearly 85% of the total Al accumulates in the cell wall of maize roots [48], while more than 70% of Al has been detected in the cell wall of wheat [39]. Among the cell wall components, pectic polysaccharides and hemicellulose are recognized as major binding sites for aluminum, with a specific emphasis on xyloglucan in hemicellulose, which has been found to be particularly sensitive to Al stress in recent studies [49,50]. Furthermore, research has revealed that one of the primary reasons for aluminum-induced inhibition of root growth is its binding to the cell wall, resulting in increased rigidity, reduced cell expansion, and diminished mechanical extensibility of the cell wall. This alteration has detrimental effects on the structure and function of the cell wall [51,52]. Theoretically, xyloglucan also plays a pivotal role in cell wall extensibility. The process of cell wall extension, which involves the cleavage of the xyloglucan backbone followed by the synthesis of a newly formed xyloglucan chain, is catalyzed by enzymes from the xyloglucan endotransglucosylase/hydrolase (XTH) family [53,54]. Our transcriptome sequencing results revealed a significant increase in the transcription levels of *XTH* upon the application of melatonin under Al stress (Supplementary Figure S2), suggesting that melatonin may upregulate the activity of XTH, which in turn regulates the content of xyloglucan, a hemicellulose capable of binding Al to reduce intracellular Al accumulation. Moreover, the alleviation of Al-induced growth inhibition in alfalfa by melatonin may be attributed to the modulation of XTH activity, thereby restoring cell wall extensibility that is otherwise reduced by Al. These results also provide us with a clear direction for an in-depth exploration of the mechanisms through which melatonin mitigates aluminum toxicity in the future.

### 3.2. Exogenous Melatonin Enhanced the Antioxidant Capacity in Alfalfa

Under normal growth conditions in alfalfa, a dynamic equilibrium exists between the generation and elimination of ROS. However, Al toxicity can disrupt this balance, leading to the excessive accumulation of ROS, which in turn, can interact with lipids and proteins, causing protein degradation and lipid peroxidation, ultimately compromising the integrity of the cell membrane [10,11,12]. Melatonin, widely known as an important animal hormone, primarily participates in regulating circadian rhythms, improving sleep quality, and combating free radicals to delay the aging process [55]. It is a highly conserved indole molecule with similar physiological functions to indole-3-acetic acid in plants, which include involvement in plant root growth, promotion of seed germination, and increasing crop yield [56]. Furthermore, numerous studies have confirmed that melatonin enhances the stress tolerance of plants. For instance, it can improve salt tolerance in rice [34], mitigate cadmium stress in Chinese cabbage [35], alleviate chromium stress in maize [36], and combat aluminum stress in soybean [38] and wheat [39], owing to its potent antioxidant capacity. In this study, we observed that the application of melatonin not only significantly reduced the H_2_O_2_ levels caused by Al but also mitigated the accumulation of MDA, an indicator of membrane lipid peroxidation, and reduced the accumulation of dead root cells, highlighting the crucial role of melatonin in scavenging ROS, alleviating membrane superoxidation, and preserving membrane integrity (Figure 3).

Plants have developed both enzymatic and non-enzymatic antioxidant systems to counteract the effects of ROS. Vital antioxidant enzymes include SOD, POD, CAT, and APX, while glutathione plays a crucial role as a non-enzymatic antioxidant in plants [57]. Our findings revealed that the application of melatonin significantly enhanced the activities of SOD, POD, CAT, and APX, thereby reducing the accumulation of H_2_O_2_ under Al stress (Figure 4), suggesting that melatonin could alleviate the imbalance in the oxidative defense system caused by Al by enhancing the activities of these enzymatic antioxidants.

### 3.3. Exogenous Melatonin Elevated C Metabolism under Al Stress in Alfalfa

The process of carbon metabolism in plants involves photosynthesis and respiration, which provides carbon compounds and serves as an energy source for various biological activities, respectively [58]. Previous studies have demonstrated that melatonin can enhance photosynthesis in various plant species under different abiotic stresses. For instance, melatonin application has been shown to improve photosynthesis in rice under salt stress [34], in tobacco under cadmium stress [59], and in maize under Al stress [40]. Given the characteristic root growth inhibition and morphological disruption observed in many plants under Al stress [4,5], we conducted a transcriptome sequencing analysis of alfalfa root tips. The results of RNA-seq analysis revealed that exogenous melatonin application significantly increased the transcript levels of enzymes related to glycolysis and gluconeogenesis in alfalfa root tips under Al stress. These enzymes include pyruvate kinase, glyceraldehyde-3-phosphate dehydrogenase, and phosphoglycerate kinase-like protein (Figure 6C). These findings suggested that melatonin might not only enhance photosynthesis in plant leaves but also improve respiration in alfalfa roots, leading to increased energy production, which could then be utilized for plant growth, thereby promoting the growth of plants exposed to Al stress.

Plant growth and carbohydrate metabolism, including sucrose and starch, are closely intertwined, as carbohydrates serve as both structural components and energy reservoirs for biomass synthesis and maintenance [60]. In higher plants, leaves (sources) are responsible for synthesizing sucrose and starch, which are subsequently transported to roots (sinks) to support root growth [60]. For instance, Zhao et al. [61] found that foliar application of melatonin significantly increased starch and sucrose synthesis in maize leaves under drought stress. Additionally, it has been reported that melatonin application could enhance the activity of sucrose phosphate synthase (SPS) and sucrose content in maize under Al stress [40]. In this present study, RNA-seq results of alfalfa roots revealed significant alterations in the starch and sucrose metabolism pathways following the addition of melatonin under Al stress (Figure 6D). Many DEGs were annotated as encoding sucrose synthase and trehalose-6-phosphate phosphatase, and they were markedly upregulated in the Al + MT vs. Al comparison. The increase in sucrose synthase transcript levels suggests a possibly elevated sucrose content in alfalfa roots, indicating that melatonin may allocate more carbohydrates to the roots under Al stress, a role consistent with its function in maize under Al stress [40]. Trehalose-6-phosphate phosphatase catalyzes the conversion of trehalose-6-phosphate to trehalose, a compound known to protect cells from various stresses and adversities in many organisms [62,63,64]. Therefore, based on the RNA-seq results, the exogenous application of melatonin might enhance the synthesis capacity of sucrose and trehalose in alfalfa, thereby improving stress resistance under Al stress. This will also be one of our main research directions in the future.

## 4. Materials and Methods

### 4.1. Plant Culture and Measurements of Al Treatments

Seeds of alfalfa were from the forage Engineering Center of Southwest University. To initiate germination, 5–6 alfalfa seeds were placed on individual moist cotton balls in a plastic container filled with 6 L of distilled water. These containers were then kept in a greenhouse under controlled conditions: 22 °C temperature, 100 μmol m^−2^s^−1^ white light intensity, and a photoperiod of 16 h of light and 8 h of darkness for one day. After three days, when two cotyledons had emerged, healthy seedlings were carefully selected to ensure that each cotton ball retained four alfalfa seedlings with uniform growth. These 4-day-old seedlings were then subjected to treatment with a 10 μM AlCl_3_ (Thermo Scientific, Waltham, MA, USA) solution containing 0.5 mM CaCl_2_ (Thermo Scientific, Waltham, MA, USA) for either 3 or 5 days at a pH of 4.2, as previously described [38]. Control groups included untreated seedlings and those treated with only 5 μM melatonin (Thermo Scientific, Waltham, MA, USA), both maintained in a 0.5 mM CaCl_2_ solution at pH 4.2. To maintain the pH at 4.2, the treatment solution was renewed daily. After the treatment period, the alfalfa seedlings were divided into two groups. The first group was used for measuring root length and fresh weight, with each treatment having four biological replicates, each containing four seedlings. The root tips from this group were used for cell anatomical observations. The second group of seedlings was separated into root and shoot sections, quickly frozen in liquid nitrogen, and stored at −80 °C for subsequent analyses, including transcriptome sequencing, Al content determination, oxidative damage indicator assessment, and enzyme activity assays.

### 4.2. Anatomical Observation of Root Tip Cells

The root tips of alfalfa seedlings, which had been treated for 3 days, were fixed in 70% FAA fixative. After fixation, they were dehydrated using a series of alcohol concentrations and embedded in paraffin. The paraffin-embedded blocks were then sectioned into 5 μm slices using a microtome. To prepare the sections for analysis, paraffin was removed with xylene and ethanol, and the slices were stained with safranin for 1 h, followed by rinsing with water. A brief immersion in fast green staining solution for 2 min followed. After dehydration with anhydrous ethanol and transparency achieved with xylene, the root tip slice images were examined under an AXIO Zoom. V16 stereo microscope (Carl Zeiss, Oberkochen, Germany) in brightfield conditions.

### 4.3. Morin Staining

Morin staining was used to assess Al accumulation, following the procedure outlined by Zhang et al. [38]. After applying various treatments, approximately 2 cm long fresh alfalfa roots were cut and immersed in 100 μM morin for 1 h at room temperature. Subsequently, the roots were washed with water to remove any excess morin. The Al–morin complex exhibited green fluorescence when excited at a wavelength of 420 nm, and the emitted light was observed at a wavelength of 510 nm.

### 4.4. Determination of Al Content

The concentrations of Al in the leaves and roots (0–1 cm) were determined using a graphite furnace atomic absorption spectrophotometer (Agilent Technologies, GTA 120 Graphite Tube Atomizer, Santa Clara, CA, USA), following the method described by Riaz et al. [65]. After the respective treatments, the alfalfa shoots and root segments (0–1 cm) were excised after three washes with distilled water. Approximately 1 g of each sample was weighed into a graphite crucible and subjected to ashing at 500 °C for 2 h to completely ash them. Subsequently, 4 g of sodium hydroxide and 1 g of sodium peroxide were added, and the mixture was melted in a muffle furnace at 650 °C for 15 min. The mixture was then heated with 50 mL of HCl to a slight boil and diluted to a final volume of 250 mL. Finally, an Al reagent was added, and the volume was adjusted to 50 mL. The spectrophotometer measured the absorbance at 530 nm. By comparing the standard curve generated using Al standard solutions of varying concentrations, the total Al content of the sample was calculated.

### 4.5. Determination of H_2_O_2_ Content

The H_2_O_2_ content in root tips was determined by measuring the formation of the titanium-peroxide complex at 415 nm using an enzyme-linked immunosorbent assay (ELISA) instrument (TECAN SPARK, Tecan Austria GmbH, Grödig, Austria) [39]. Briefly, we added 1 mL of acetone to 0.1 g of tissue. The mixture was then homogenized in an ice bath and centrifuged, after which 2.0 mL of a solution containing 20% sulfuric acid (*v*/*v*) and 0.1% titanium tetrachloride (*v*/*v*) was added to the 200 μL supernatant. The supernatants obtained after centrifugation were analyzed at 415 nm using the ELISA instrument (TECAN SPARK, Tecan Austria GmbH, Grödig, Austria). The H_2_O_2_ concentration in the samples was determined by comparing the absorbance values to a standard curve of known H_2_O_2_ concentrations.

### 4.6. Analysis of Oxidative Damage

To assess lipid peroxidation, the levels of 2-thiobarbituric acid-reactive metabolites (TBA) were measured, with these TBA-reactive substances quantified as malondialdehyde (MDA), which serves as an end product of lipid peroxidation [66].

Firstly, 1 mL of extraction solution was added to approximately 0.1 g of root tips. The mixture was homogenized in an ice bath and then centrifuged at 4 °C and 12,000 rpm for 10 min. Next, 300 μL of a reaction solution containing 0.5% thiobarbituric acid (TBA) was added to 200 μL of the supernatant, followed by incubation at 95 °C for 30 min. After centrifugation, the MDA content was determined and calculated by measuring the absorbance at 532 nm and 600 nm using an ELISA instrument (TECAN SPARK, Austria). The integrity of the plasma membrane was assessed using a 0.25% Evans blue solution, which is capable of entering dead cells [66]. The root apices were immersed in a solution of 0.25% Evans blue dye for 20 min and then thoroughly washed with water 3–5 times. Finally, the root tips were examined using a microscope under brightfield conditions.

### 4.7. Determination of Antioxidant Enzyme Activity

For enzyme activity assays, approximately 0.1 g of frozen root samples were homogenized and extracted in PBS buffer, followed by centrifugation at 4 °C and 12,000 rpm for 10 min. Subsequently, the supernatant was collected via pipetting for enzyme activity determination. The activity of superoxide dismutase (SOD; EC 1.15.1.1) was assessed using the total superoxide dismutase assay kit with WST-8 (Geruisi, Suzhou, China) [67]. In the coupled reaction system of WST-8 and xanthine oxidase (XO), the SOD enzyme activity in the reaction system was defined as one unit of enzyme activity (U/mL) when the inhibition percentage reached 50%. The activity of peroxidase (POD; EC 1.11.1.7) was determined by monitoring the oxidation of guaiacol upon the addition of H_2_O_2_ [39]. One unit (U) of POD activity was defined as the increase of 0.5 in absorbance at 470 nm per minute per gram of root sample in the reaction system. The activity of catalase (CAT; EC 1.11.1.6) was measured by observing the decrease in absorbance at 240 nm, reflecting the reduction in H_2_O_2_ concentration [66]. One unit (U) of CAT activity was defined as the degradation of 1 μM H_2_O_2_ per minute per gram of root sample. The activity of ascorbate peroxidase (APX; EC 1.11.1.11) was quantified by measuring the decrease in absorbance at 290 nm, which resulted from the oxidation of ascorbic acid by the complex of APX and H_2_O_2_ [65]. The unit (U) of APX activity was defined as the oxidation of 1 μM of ascorbate per minute per gram of root sample.

### 4.8. RNA Extraction and Transcriptome Sequencing

After 3 days of treatment with Al alone or in combination with melatonin, we collected 2 cm of alfalfa roots for transcriptome sequencing. The groups treated only with acid and only with melatonin were used as controls. Each treatment was replicated three times. Total RNA from each sample was extracted using the Polysaccharide Polyphenol RNA Extraction Kit (QIAGEN, Hamburg, Germany), following the manufacturer’s instructions. The concentration and integrity of RNA were assessed using the Nanodrop spectrophotometer (Thermo Scientific, Waltham, MA, USA) and the 2100 bioanalyzer (Agilent Technologies, Santa Clara, CA, USA), respectively. Samples with RNA integrity >8 and OD260/280 >8 were selected for library construction [68]. Subsequently, the library was constructed using the NEBNext Ultra RNA Library Prep Kit for Illumina (NEB, Ipswich, MA, USA). The concentration and insert size of the library were detected using the Qubit 2.0 Fluorometer and the Agilent 2100 Bioanalyzer (Agilent Technologies, Santa Clara, CA, USA), respectively. Once the concentration and insert size met the expected range, Q-PCR was performed to accurately quantify the effective concentration of the library, ensuring that the library’s effective concentration was higher than 2 nM to ensure library quality. Finally, the libraries were sequenced using the Illumina HiSeq platform (Novogene, Beijing, China) [40].

### 4.9. Differential Expression Analysis

We used the DESeq2 R package (1.20.0) for differential expression analysis [69]. DESeq2 uses statistical methods to identify differential expression in digital gene expression data by employing a model that is based on the negative binomial distribution. The obtained *p*-values were adjusted using the Benjamini and Hochberg method to control the false discovery rate. Based on the results, we screened for DEGs using the criteria: “|log2fold change| ≥ 1 and adjusted *p*-value ≤ 0.05”. 

### 4.10. GO and KEGG Enrichment Analysis of DEGs

The clusterProfiler R package was used to perform gene ontology (GO) enrichment analysis on differentially expressed genes, with a correction made for gene length bias [70]. GO terms with adjusted *p*-value < 0.05 were considered significantly enriched by differentially expressed genes. The Kyoto Encyclopedia of Genes and Genomes (KEGG) (http://www.genome.jp/kegg/ accessed on 30 November 2022) is a comprehensive database resource that integrates data on genomic, chemical, and phylogenetic functions [71]. We utilized the clusterProfiler R package to assess the statistical enrichment of differentially expressed genes in KEGG pathways.

## 5. Conclusions

In this study, we conducted a comprehensive physiological and transcriptomic analysis to determine the mechanisms underlying melatonin’s ability to alleviate Al toxicity in alfalfa. Our results demonstrated that melatonin could effectively mitigate the negative effects of Al on alfalfa growth, including growth inhibition and damage to root tip cells, which was associated with a reduction in Al accumulation in both root and shoot tissues. Additionally, melatonin played a role in scavenging excess H_2_O_2_, reducing lipid peroxidation as indicated by decreased MDA levels, and enhancing the activities of antioxidant enzymes such as SOD, POD, and CAT. Our RNA-seq data revealed that melatonin treatment upregulated the expression of genes involved in key processes such as cell wall, glycolysis, and sucrose and starch metabolism, suggesting that melatonin might potentially alleviate Al toxicity through mechanisms such as enhancing the binding of aluminum to the cell wall, improving respiration, and increasing the content of sucrose. These molecular and physiological changes collectively contribute to the alleviation of Al toxicity in alfalfa. Taken together, our research provides valuable insights into the mechanisms through which melatonin mediates aluminum stress in alfalfa, and the findings hold the potential to inform strategies for improving alfalfa production in acidic soils, which could benefit agriculture and forage production.

## Data Availability

Data are contained within the article or Supplementary Material.

## Supplementary Materials

The following supporting information can be downloaded at: https://www.mdpi.com/article/10.3390/ijms242417221/s1.
