# Supplementary material for: Physiological and Transcriptomic Analysis Reveals That Melatonin Alleviates Aluminum Toxicity in Alfalfa (Medicago sativa L.)
Source: Int J Mol Sci. 2023 Dec 7;24(24):17221. doi: 10.3390/ijms242417221 (PMC10743983; doi:10.3390/ijms242417221)
Supplement: Supplementary file 1 [file ijms-24-17221-s001.zip › ijms-2724712-SI.pdf]

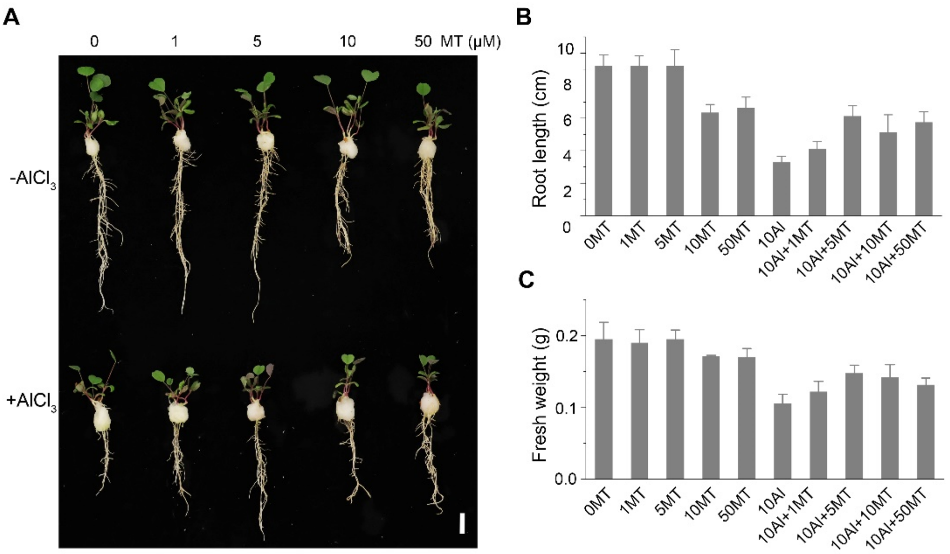

Supplementary Figure S1: Effects of different concentrations of melatonin on the phenotype of alfalfa. (A) Phenotypes after 5d of $AlCl_3$ and melatonin treatment. Scale bar, 1cm. (B) Root length, data were means ± SE n=16. (C) Fresh weight of 4 alfalfa seedlings on a cotton ball, data were mean ± SE n=4.

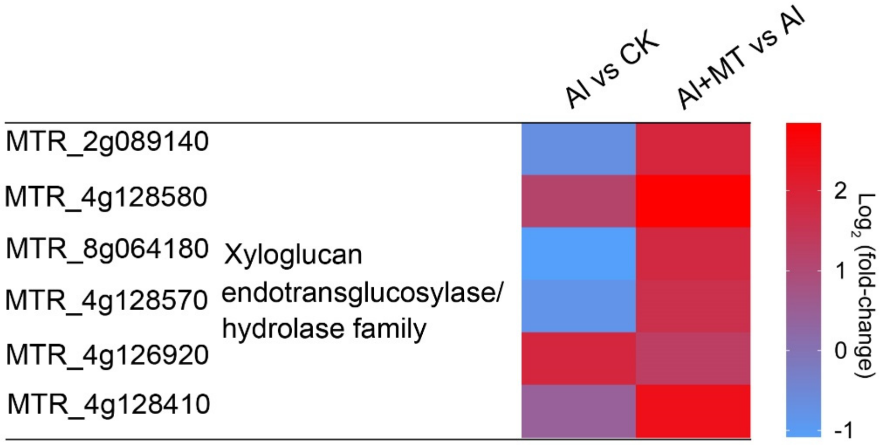

Supplementary Figure S2: Transcript changes in cell wall-related genes induced by Al and melatonin.

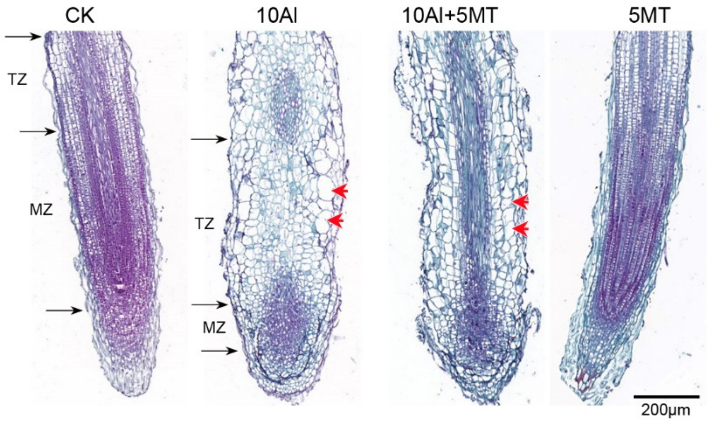

Supplementary Figure S3: The longitudinal section of alfalfa root apex, using safranin and fast green dyes for staining. meristematic zone (MZ), transition zone (TZ). The areas between the two black arrows represent the meristematic zone (MZ) and the transition zone (TZ). The cells indicated by the red arrows represent significant changes in the size and shape of transition zone cells after different treatments.
